# Renalase and hypertension—demographic and clinical correlates in obstructive sleep apnea

**DOI:** 10.1007/s11325-020-02157-3

**Published:** 2020-08-06

**Authors:** Helena Martynowicz, Karolina Czerwińska, Anna Wojakowska, Lidia Januszewska, Iwona Markiewicz-Górka, Mieszko Więckiewicz, Grzegorz Mazur, Krystyna Pawlas, Rafał Poręba, Paweł Gać

**Affiliations:** 1 Department of Internal Medicine, Occupational Diseases, Hypertension and Clinical Oncology, Wroclaw Medical University, 213 Borowska St., 50-556 Wroclaw, Poland; 2 Department of Hygiene, Wroclaw Medical University, 7 Mikulicza-Radeckiego St., 50-345 Wroclaw, Poland; 3 Department of Experimental Dentistry, Wroclaw Medical University, 26 Krakowska St., 50-425 Wroclaw, Poland

**Keywords:** Renalase, Obstructive sleep apnea, Hypertension

## Abstract

**Background:**

Renalase plays an important role in blood pressure regulation. Obstructive sleep apnea (OSA) is a common respiratory disorder associated with hypertension and cardiovascular complications. The aim of the study was to assess the relationship between sleep apnea and renalase concentration.

**Material and methods:**

Adult patients (*n* = 113) were evaluated for OSA in a sleep laboratory using polysomnography. The respiratory events were scored according to the standards developed by the American Academy of Sleep Medicine. The blood renalase concentration was determined by the ELISA (enzyme-linked immunosorbent assay) test.

**Results:**

OSA (AHI ≥ 5) was diagnosed in 71% (*n* = 80) of the studied population. Renalase concentration was statistically significantly lower in the group with moderate-to-severe OSA (AHI ≥ 15) compared with the group without OSA (AHI < 5) (139.56 ± 175.72 ng/ml vs. 230.97 ± 240.50 ng/ml, *p* = 0.042). We have found statistically significant negative correlation between renalase and AHI in hypertensives, but not in normotensives. The statistically significant negative correlation was observed between AHI and renalase in the whole studied group, in males, and in the group of age < 60 years old. There was not such a correlation in females and in the group > 60 years old. Based on the regression model, it was shown that lower renalase concentration, hypertension, higher BMI, and male gender are independently associated with higher AHI.

**Conclusions:**

There is a relationship between the blood renalase concentration and the severity of OSA, which may influence hypertension development in OSA.

## Introduction

Obstructive sleep apnea (OSA) is a common disorder characterized by recurrent episodes of either complete upper airway collapse (apneas) or partial collapse (hypopneas) during sleep leading to arterial oxygen desaturation, frequently terminated by arousal [1]. OSA is independently associated with cardiovascular morbidity and mortality [2–5]. The meta-analysis determined that severe OSA (apnea/hypopnea index ≥ 30) was associated with increased cardiovascular mortality with a hazard ratio of 2.73 [6]. The parameter that defines the severity of OSA is the apnea/hypopnea index (AHI), which indicates the number of episodes per hour. OSA is diagnosed if an AHI ≥ 5 events per hour. AHI between 5 and 15 indicates mild OSA, between 15 and 30—moderate OSA, and more than 30—severe OSA [7]. It was demonstrated that about 50% of patients with OSA have underlying arterial hypertension [7, 8]. Recent meta-analysis, involving 20 original studies in 19 articles, demonstrates that OSA increases the risk of hypertension in a dose-response manner [9]. The pathogenesis of hypertension in OSA are complex and not fully understood. The main risk factors for OSA are older age, male gender, and obesity [10]. It has been suggested that intermittent hypoxemia, sleep fragmentation, nocturnal fluid shift, activation of renin-angiotensin-aldosterone (RAA) system, and endothelial dysfunction may play a role in increase of blood pressure. During OSA episodes, there may be an upregulation of the sympathetic nervous system which, through chemoreceptor reflexes, can cause hypertension [11, 12]. Meta-analysis on OSA treatment with CPAP treatment trials found a low reduction of blood pressure (2.6 mmHg for systolic and 2.0 mmHg for diastolic blood pressure) [12]. Thus, CPAP does not seem to be the effective in blood pressure reduction and novel and more effective methods of treatment are needed.

Renalase, one of the recently discovered flavoproteins, is possibly an important determinant of cardiovascular health. Renalase has been shown to be responsible for the elimination of circulating catecholamines in the plasma [13–15]. Catecholamines are involved in sympathetic activation; thus, renalase may play a crucial role in pathogenesis of arterial hypertension [16]. Desir et al. showed that the decrease of renalase gene expression about 40% if followed by the blood pressure increased by 13 mmHg [16]. At least two single nucleotide polymorphisms of the renalase gene have been documented as risk factors for the development of hypertension [17]. The increased renalase has been reported in patients with arterial hypertension [16] and positively related to blood pressure in clinical studies [18, 19]. Recently, the relationship between blood renalase concentration and the intensity of sleep bruxism has been shown [20].

The aim of the study was to assess the relationship between sleep apnea and blood renalase concentration as a possible mechanism of the development of arterial hypertension in patients with OSA.

## Material and methods

**Fig. 1:**
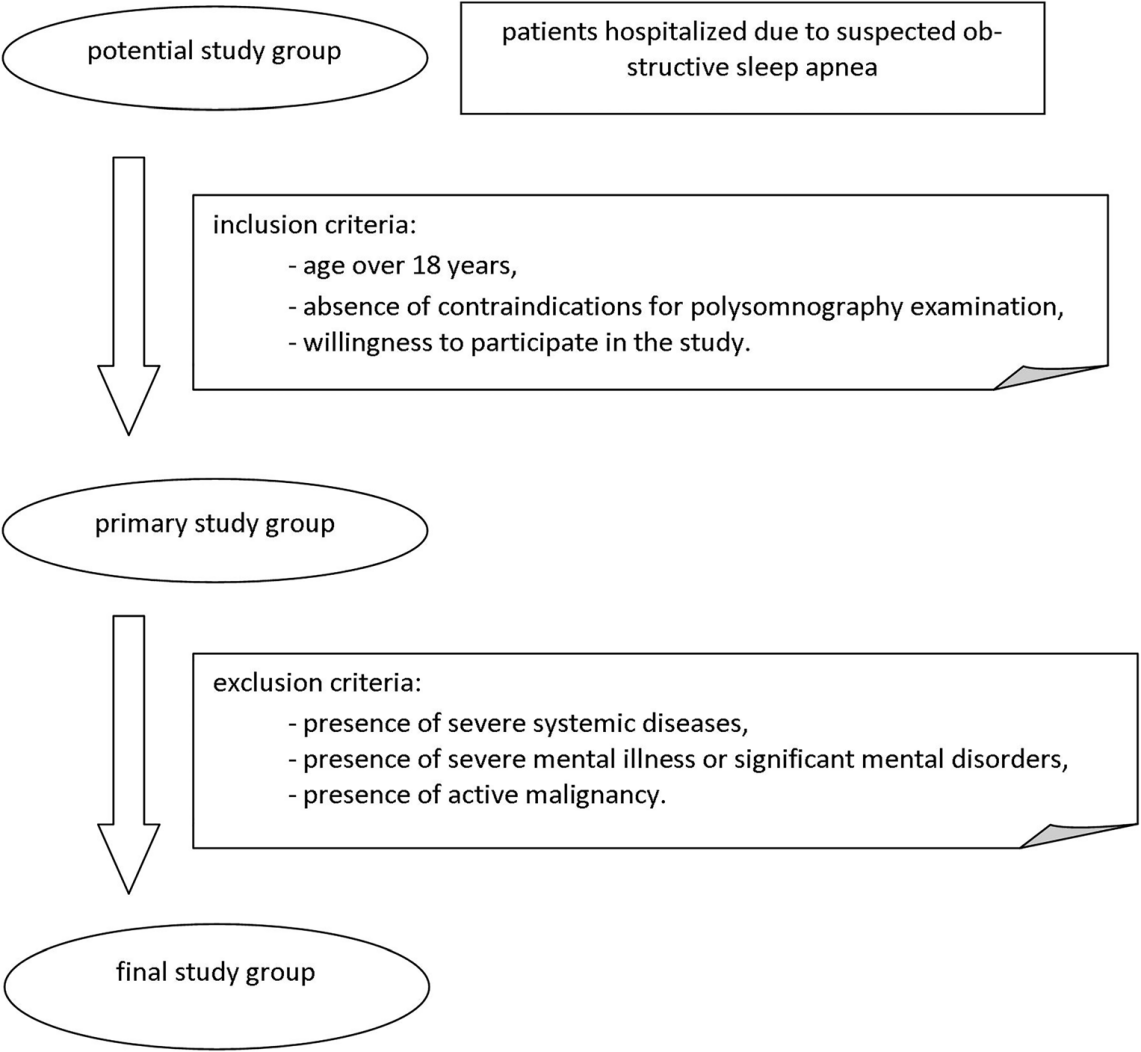
The qualification scheme for recruitment for the research project

The research was carried out on a group of 113 adult patients hospitalized in an internal medicine clinic to verify the suspicion of OSA. The criteria for qualifying patients for the study were as follows: consent to participate in the study, age ≥ 18 years, and no contraindications for polysomnography (PSG). The criteria for disqualification from the study were as follows: coexistence of severe systemic diseases, coexistence of severe mental illness/mental disorders, and coexistence of active proliferative disease. The qualification scheme for recruitment for the research project is shown in Fig. 1.

PSG was performed according to a diagnostic standard as a nocturnal, single-night recording, using the Nox-A1 machine (Nox Medical, Iceland). The PSG recordings included electroencephalography, electromyography, electrooculography, pulse oximetry, electrocardiography, and a microphone. The airflow was measured using a nasal pressure transducer, and the respiratory effort of thoracoabdominal movement was measured using respiratory inductance plethysmography. Polysomnograms were assessed in 30-s epochs according to the typical criteria for assessing the quality of sleep developed by the well-known American Academy of Sleep Medicine (AASM) [21]. The following polysomnographic parameters were assessed: total sleep time (min), sleep latency (min), sleep efficiency (%), and the percentages of duration of subsequent sleep phases: N1, N2, N3, and REM. The AHI was defined as the average number of episodes of apnea and hypopnea per hour of total sleep time (TST). Apnea was attained with the reduction of air flow to less than 10% of the baseline for at least 10 s. A hypopnea episode was defined as a decrease in the nasal pressure signal by at least 30% from baseline for at least 10 s, with a reduction in O_2_ saturation of at least 3% from the pre-event baseline or an arousal.

Blood was collected in the morning after polysomnography, usually by puncturing the veins of the ulna. Until renalase determinations were performed simultaneously in all samples, the blood was stored at a constant temperature. Serum renalase determinations were performed using the E3109Hu kit ELISA (enzyme-linked immunosorbent assay) (Bioassay Technology Laboratory, Shanghai, China). The determinations were made strictly according to the test manufacturer’s instructions. The renalase concentration was expressed in the unit nanogram per milliliter. The reference range of the assay used was 1–400 ng/ml. According to the manufacturer, the sensitivity of the ELISA test used was 0.52 ng/ml. The coefficient of intra- and inter-assay variation was < 8% and < 10%.

Statistical analyses were conducted using Dell Statistica 13 software (Dell Inc., USA). The quantitative variables were expressed as means and standard deviations. The qualitative variables were expressed as percentages. The distribution of variables was tested with the W-Shapiro-Wilk test. In the case of the quantitative variables of normal distribution, a further statistical analysis used *T* test. For non-normally distributed quantitative variables, the Mann-Whitney *U* test was used. For qualitative variables, in further statistical analysis, the chi-square test of maximum likelihood was used. To determine the relation between the examined variables, the correlation and regression analysis has been conducted. In the case of normal distribution, the Pearson correlation *r* factors have been determined whereas in the case of non-normal distribution, the Spearman *r* factors have been applied. The possible factors related to AHI value were found on the basis of the univariable linear regressions between AHI value and basic anthropometrical data (age, BMI, gender), cardiovascular diseases (hypertension, coronary artery diseases, type 2 diabetes), and also renalase concentration. Then, using multivariable stepwise regression analysis, taking into account statistically significant factors demonstrated in univariate analyses, the final model for the AHI value was obtained. The parameters of the models obtained in the regression analysis have been estimated by the application of the least square’s method. Results with *p* < 0.05 were considered statistically significant.

The studies were carried out as part of a research project entitled “The importance of relationship between selenium and zinc deficiency and blood renalase concentration in the pathogenesis of subclinical cardiovascular complications of hypertension,” according to the records in the Simple system with the number SUB.A100.19.009, which obtained the consent of the local Bioethics Committee.

## Results

The mean age of all participants was 49.70 ± 14.60 years. Women constituted 53% (*n* = 60) of all participants. The mean BMI was found to be 28.71 ± 5.3 kg/m^2^. Diabetes and ischemic heart disease were diagnosed in 9.7% (*n* = 11) and 7.07% (*n* = 8) of the study population, respectively. Thirty-eight percent (*n* = 43) were diagnosed as hypertensives.

OSA (AHI ≥ 5) was diagnosed in 71.42% (*n* = 80) of the studied population. The mild (AHI 5–15), moderate (AHI 15–30), and severe (AHI ≥ 30) OSA were diagnosed in 28.57% (*n* = 32), 18.75% (*n* = 21), and 24.10% (*n* = 27) of the study population, respectively. The mean apnea/hypopnea index (AHI) was 17.98 ± 18.82. The polysomnographic parameters in the studied group are presented in Table 1.

**Table 1:** Parameters of polysomnographic examination in the examined group (*n* = 113)

Parameter	Mean ± SD	Minimum	Maximum
SE	81.80 ± 10.08	52.40	98.30
SL	21.38 ± 21.20	0.00	112.60
WASO	56.63 ± 43.73	1.00	186.00
N1 (%)	5.01 ± 4.23	0.30	21.20
N2 (%)	46.01 ± 11.73	4.30	78.70
N3 (%)	26.70 ± 11.21	2.60	54.90
REM (%)	21.89 ± 7.62	4.10	38.40
AHI	17.98 ± 18.82	0.00	87.40
OAI	5.67 ± 11.65	0.00	71.20
MAI	0.19 ± 1.40	0.00	14.80
CAI	0.76 ± 2.55	0.00	17.60
Ch-S R	0.85 ± 2.76	0.00	16.80
Min SatO_2_	83.49 ± 8.03	54.00	95.00
Mean SatO_2_	93.21 ± 2.46	83.30	97.30
ODI	17.22 ± 18.25	0.00	88.40

*SE* sleep efficiency, *SL* sleep latency, *WASO* wake after sleep onset, *REM* rapid eye movement, *AHI* apnea-hypopnea index, *OAI* obstructive apnea index, *MAI* mixed apnea index, *CAI* central apnea index, *Ch-S R* Cheyne-Stokes respiration, *MinSatO*_*2*_ minimal oxygen saturation, mean *SatO*_*2*_ mean oxygen saturation, *ODI* oxygen desaturation index

The mean concentration of renalase was 185.61 ± 213.38 ng/ml in the entire study group. There was no statistically significant difference in renalase concentration in the group with OSA (AHI ≥ 5) and controls without OSA (AHI < 5) (169.15 ± 200.98 ng/ml vs. 230.97 ± 240.50 ng/ml, *p* = 0.168). However, renalase concentration was statistically significantly lower in the group with moderate-to-severe OSA (AHI ≥ 15) compared with the group without OSA (AHI < 5) (139.56 ± 175.72 ng/ml vs. 230.97 ± 240.50 ng/ml, *p* = 0.042). Furthermore, renalase concentration was statistically significantly lower in the group with moderate-to-severe OSA (AHI ≥ 15) compared with the group with mild-to-no OSA (AHI < 15) (139.56 ± 175.72 ng/ml vs. 221.76 ± 233.23 ng/ml, *p* = 0.044).

**Fig. 2:**
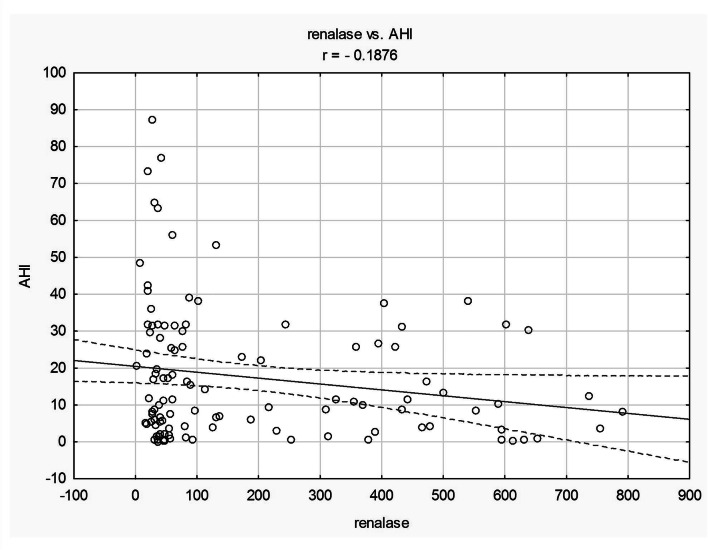
The correlation between renalase (ng/ml) and AHI in whole studied group

The statistically significant negative correlation was observed between AHI and renalase in the entire study group (*r* = − 0.19, *p* = 0.039; Fig. 2), in males (*r* = − 0.29, *p* = 0.046), and in the group of age < 60 (*r* = − 0.20; *p* = 0.042). There was not such a correlation in females and in the group > 60 years old. The statistically significant negative correlation was also observed between AHI and renalase in hypertensives and in the group with OSA (AHI ≥ 5) (*r* = − 0.22, *r* = − 0.17, respectively). There was not such a correlation in normotensives and in healthy controls (AHI < 5). We have also observed statistically significant negative correlation between renalase and oxygen desaturation index (ODI) (*r* = − 0.18, *p* = 0.041) and between renalase and mean desaturation (*r* = − 0.18, *p* = 0.039) in the entire study group.

Then, we divided the study group according to the following criteria: first quartile, median, and third quartile renalase concentration. Statistically significant differences were obtained only in comparison of subgroups separated by 1 quartile of renalase concentration. We have observed increased AHI, mean oxygen saturation (SatO_2_), minimal oxygen saturation, stage N1 sleep duration, central apnea index, and Cheyne-Stokes breathing duration in the group with renalase concentration < first quartile compared with the group with renalase concentration ≥ first quartile (renalase < 36.43 ng/ml vs. renalase ≥ 36.43 ng/ml) (Table 2).

**Table 2:** Parameters of polysomnographic examination in studied group divided due to first quartile of renalase concentration (with renalase concentration < 36.43 ng/ml and renalase ≥ 36.43 ng/ml)

	Renalase < 36.43 ng/ml	Renalase ≥ 36.43 ng/ml	*p*
SE	83.98 ± 9.42	81.20 ± 10.30	0.239
SL (min)	15.44 ± 12.72	23.27 ± 15.79	0.114
WASO	56.44 ± 51.28	53.11 ± 41.75	0.746
N1 (%)	4.64 ± 3.48	5.12 ± 4.47	0.632
N2 (%)	50.54 ± 13.68	44.70 ± 10.90	0.032*
N3 (%)	23.92 ± 11.02	27.46 ± 11.26	0.177
REM (%)	20.90 ± 9.26	22.21 ± 7.14	0.464
AHI	24.14 ± 23.22	15.35 ± 15.76	0.028*
OAI	7.72 ± 12.29	4.23 ± 8.90	0.111
MAI	0.64 ± 2.83	0.04 ± 0.16	0.053
CAI	1.90 ± 4.58	0.41 ± 1.25	0.008*
Ch-S R (%)	2.02 ± 4.85	0.48 ± 1.47	0.012*
Min SatO_2_	80.65 ± 8.90	84.73 ± 6.85	0.015*
Mean SatO_2_	92.21 ± 2.34	93.64 ± 2.16	0.004*
ODI	21.72 ± 21.80	15.14 ± 15.74	0.093

Mean ± SD

*SE* sleep efficiency, *SL* sleep latency, *WASO* wake after sleep onset, *REM* rapid eye movement, *AHI* apnea-hypopnea index, *OAI* obstructive apnea index, *MAI* mixed apnea index, *CAI* central apnea index, *Ch-S R* Cheyne-Stokes respiration, *MinSatO*_*2*_ minimal oxygen saturation, *mean SatO*_*2*_ mean oxygen saturation, *ODI* oxygen desaturation index

*Statistically significant

The correlation analysis showed the existence of statistically significant relationships between AHI on the one hand and male sex, BMI, age, hypertension, and ischemic heart disease on the other (*r* = 0.41, *r* = 0.30, *r* = 0.41, *r* = 0.37, *r* = 0.31; respectively).

With the use of a multivariable stepwise regression analysis of statistically significant variables from univariate regressions (male sex, BMI, age, hypertension, coronary artery diseases, and renalase concentration), a final model was obtained with the AHI as the dependent variable:

$$ \mathrm{AHI}=-2.966-0.007\ \mathrm{renalase}+5.605\ \mathrm{hypertension}+0.644\ \mathrm{BMI}+11.268\ \mathrm{male}\ \mathrm{sex} $$

Based on the regression model that was obtained, it was shown that lower renalase concentration, hypertension, higher BMI, and male sex are independently associated with higher AHI values (Table 3).

**Table 3:** Univariate and multivariable stepwise regression models determining factors related to AHI values in the study group

	Model for: AHI				
	Univariate regression		Multivariable regression		
	Rc	*p*	Rc	SEM of Rc	*p*
Intercept	-	-	− 2.966	2.924	0.042*
Age (years)	0.535	0.001*	0.181	0.157	0.253
BMI (kg/m^2^)	1.119	0.010*	0.644	0.393	0.011*
Male sex	15.570	0.001*	11.268	4.039	0.007*
Coronary artery diseases	22.641	0.001*	9.937	6.913	0.155
Type 2 diabetes	0.518	0.934	-	-	-
Hypertension	14.454	0.002*	5.605	4.430	0.021*
Renalase (ng/ml)	− 0.016	0.039*	− 0.007	0.009	0.047*

*AHI* apnea/hypopnea index, *BMI* body mass index

*Statistically significant

## Discussion

The most important result of this study is the finding of an inversely proportional linear relationship between renalase concentration and AHI values in the entire study group. The result indicates association between OSA and renalase, which is an enzyme involved in hypertension development. We have also observed linear negative correlations between renalase concentration and mean oxygen desaturation, but not mean oxygen saturation (SatO_2_). This result indicates role of variability of blood oxygen saturation more than stable hypoxemia in OSA pathomechanism.

Then, we have divided the study group into subgroups: group with AHI ≥ 15 and group with AHI < 15, since moderate-to-severe OSA (AHI ≥ 15) is associated with increased cardiovascular risk [22]. We have found decreased renalase concentration in the group with moderate and severe OSA (AHI ≥ 15) compared with the group with AHI < 15. In patients with moderate-to-severe OSA compared with mild OSA, the risk of hypertension is also significantly higher [23]. Recently Hou confirmed association between moderate and severe OSA and hypertension, but not mild OSA [23]. In the study, we have observed negative linear correlation between renalase concentration and AHI in hypertensives, but not in normotensives. Since renalase is known as essential hypotensive factor, decreased levels of the enzyme might lead to hypertension development. Thus, the result of the study agrees with previous studies and might indicate the role of renalase in hypertension pathogenesis due to sleep apnea.

It is noteworthy that some studies raised the relationship between OSA and hypertension in a gender. Recently, meta-analysis showed that men suffer from higher incidence of essential hypertension than women, as well the association in Caucasians is greater than that in Asians [9]. It was also demonstrated that an RDI (respiratory disturbance index) ≥ 14 was associated with a increased risk for hypertension for men. This association was not statistically significant among women [24]. Interestingly, we have also observed correlation between renalase and AHI in males. However, there was no correlation between renalase and AHI in females. The results suggest an association between OSA and hypertension occurring in men but not in women in both studies.

We have observed correlation between renalase and AHI in group < 60 years old, but not in the group > 60 years old. The recent review of the overall body of evidence confirms that advancing age, male sex, and higher body mass index increase OSA prevalence [25]. The data concerning influence of age on hypertension development in OSA are limited. The prevalence of hypertension increases with age; however, this result may indicate different mechanisms of hypertension in older age. Due to present study, renalase is correlated with OSA in younger participants and consequently may be involved in hypertension pathogenesis in age ranges < 60 years old.

We have found increased stage N2 sleep duration in the group with low renalase concentration (in the first quartile of renalase concentration < 36.43 ng/ml) compared with the group with higher renalase concentration (> 36.43). This result indicates worse sleep quality in patients with low renalase concentration. Increased duration of light sleep stages accompanied with decreased deep stages of sleep was observed in sleep apnea [26]. We have observed increased AHI in the group with low renalase concentration, which agrees with these studies. We have also found increased central apnea index and Cheyne-Stokes respiration duration in the group with low renalase concentration. Cheyne-Stokes respiration occurs with CSA (central sleep apnea) and is characterized by cyclical crescendo-decrescendo respiratory effort that occurs without upper airway obstruction [27, 28]. Cheyne-Stokes breathing in CSA is usually more common during the NREM sleep phase, especially during periods N1 and N2 [29].

There was a negative correlation between renalase and AHI in the group with OSA (AHI > 5), but not in healthy controls (AHI < 5), which may suggest the involvement of renalase in OSA pathomechanism. We have also confirmed relationship between AHI and male gender, BMI, age, hypertension, and ischemic heart disease. Based on the segment multifactorial regression analysis performed in the entire study group, we have found that lower blood renalase concentration, hypertension, higher BMI, and male gender are independently associated with higher AHI. The association between OSA and hypertension [30], BMI [31], age [32], and male gender [33] was showed in numerous previous studies. To our knowledge, it is the first study which demonstrated relationship between OSA and renalase. The results of the study might indicate the role of renalase in OSA and comorbid hypertension pathogenesis.

The main disadvantage of this study is the absence of both office and ambulatory blood pressure monitoring values for participants. We have neither asses the level of catechalamine’s. The gender distribution in the studied group differs from the typical one—we have over-representation of women.

In summary, we showed a relationship between blood renalase concentration and obstructive sleep apnea. It is worth noting that recently our research team demonstrated the role of renalase in sleep bruxism [20]; thus, renalase holds promise as a potential important factor of hypertension pathomechanism during sleep in both sleep bruxism and obstructive sleep apnea.
